# Abstracts from Foreign Journals

**Published:** 1901-12

**Authors:** M. P. Ravenel, S. J. J. Harger

**Affiliations:** Philadelphia, Pa.; Philadelphia, Pa.


### ABSTRACTS FROM FOREIGN JOURNALS.

### FRENCH.

Under the Direction of M. P. Ravenel, M.D., and S. J. J. Harger, V.M.D., of Philadelphia, Pa.

Arterio-sclerosis in the Spinal Cord. Subject, horse, aged eight years. General emaciation, affecting especially the muscles of the back and croup ; hydropsy of the stifle-joint. The posterior members, during walking, were stiff, not flexed, dragged and abducted; at a trot, and while turning, there was evident loss of muscular coördination. The animal could trot or gallop only for a short distance.

The lesion of the stifle had existed since the horse was four years old, and Watson diagnosed a lesion of the spinal cord and especially of the superior columns.

The post-mortem revealed a chronic arthritis of the stifle, back, and sacro-iliac articulation. Microscopical examination of the spinal cord showed in the dorsal region a diminution (degeneration) of a number of nerve fibres in the posterior columns, a narrow cavity in the gray substance on one side, and a diffuse arterio-sclerosis, most marked in the posterior columns; similar lesions in the cervical region, with numerous small, irregular cavities. These lesions were most marked in the lumbar region, along the course of the origin of the sensitive roots of the spinal nerves. This vascular sclerosis caused a narrowing and obliteration of the bloodvessels, which explained the degeneration of the nerve fibres and the cavity-like softening of the cord in the areas receiving an insufficient blood-supply.

The author inquires if the more extensive lesions of the superior portion of the spinal cord are due to the fact that certain toxins have a greater elective action upon the vessels of this region.—*The Veterinary Journal.*

Etiological Agent of Vaccine and Variola. The following article is the work of Dr. Funck, chief of the Bacteriological Laboratory of the University of Brussels, whose experiments upon this point have extended over a period of two years.

1. *Vaccinia is not a microbic disease.* It has been known for a long time that freshly prepared vaccine—a glycerinated emulsion of the contents of the vaccine pustules—contains a large number of bacteria. The vaccinal lymph is obtained from the scabs of the pustules—a process which cannot be performed in an aseptic manner.

This vaccine undergoes autopurification, and after three months most of the accidental microbes primitively in the lymph will disappear. Twenty different samples have been studied, being kept in sealed tubes and not exposed to light, and after three months they were sterile (free from bacteria), although they still produced characteristic pustules.

2. *The etiological agent of vaccine is a protozoön.* If a microscopical preparation of the vaccine be made in the ordinary way, drying it on the glass slide and staining warm or cold, a large number of clear and transparent vacuoles not staining with aniline will be seen. A microscopical examination (500 to 600 diameters) of a preparation of fresh vaccine fixed by heat will show that the clear spaces (vacuoles) are very characteristic morphological elements which have previously been mentioned by several observers, especially L. Pfeiffer, in 1887.

These bodies most frequently exist under three different forms:

*a.* Regularly round, with a brilliant green reflection, slow movements when the slide is heated to 37° C., measuring 2 to 10$\mu$.

*b.* Small, ovoid cells, with a lateral nucleus, whose protoplasm contains small, bright green spheres analogous to the preceding elements, and measuring 1 to 3$\mu$ in diameter. These are the epidermic cells infected by the protozoön.

*c.* Finally, a raspberry mass (morula), sometimes round, with or without a double contour, measuring 25$\mu$ in diameter, sometimes oval, 30 to 35$\mu$ long and 20 to 25$\mu$ in diameter. These elements are the sporoblasts containing the spores, which show a large, clear nucleus, sometimes central, sometimes lateral. At times they are pyriform in shape—round at one end and pointed at the other; they are frequently found in the pustules in tubular masses containing 20 to 40 of these bodies. The last form is found most frequently in old vaccine, while in the fresh lymph the first form predominates. The author designates this organism *sporidium vaccinale.*

An emulsion of the spores was made with a drop of bouillon and inoculated into a calf. Toward the sixth day characteristic pustules formed. Calves which were inoculated with fresh emulsions of this protozoön were immune to subsequent vaccinations, and not the least local reaction was produced.

Funck examined the contents of the vesico-pustules of several cases of confluent variola; they contained the same elements as were found in the vaccinal pustule.

Having been able to produce variola in calves and, after three or four passages upon this animal, to obtain a vaccine (Hacclus and Hime), the author concludes that the causative agent of vaccine and variola are identical—the parasitic element probably being a protozoön.—*Ann. de Méd. Vét.*

Treatment of Distemper. Döttl recommends the daily administration of 1 gramme of sulphonal in cases complicated with paralysis of the hind legs; even when the hind legs are dragged along the ground, he has found that sulphonal effects many cures without other treatment. This drug is also used in cerebral convulsions. The abortive treatment of the disease at its beginning consists in the administration of calomel. —*Thierärztliches Centralblatt.*

Treatment of Toe-crack. There are three principal indications in the treatment of toe-crack:

1. Immobilization of the borders of the seam.
2. Stimulation of the horn secretion.
3. No wall-bearing surface for the shoe in the region of the crack.

The first indication is the principal one. Professor Pench, as well as others, found nothing better than the old way of clasping the crack with a horseshoe nail. A horizontal hole, beginning about an inch from the crack, is drilled through the wall and a long nail inserted and clinched.—*Rec. de Méd. Vét.*

Protective Vaccination in Swine Fever. This is permitted in Spain only under the most severe restrictions, the disease being treated with the same comprehensive regulations as cattle plague. The dose of serum as prepared at the Pasteur Institute is 10 c.c. for pigs under 50 kilos and 20 c.c. over 50 kilos. The injection is given to all animals exposed to infection at the time of outbreak, and as a curative measure is repeated in 10 c.c. doses every six or twelve hours. The period of immunity conferred is considered to be from five to twelve months. Owing to the various results recorded the procedure is to be considered as purely experimental, and sanitary regulations must be depended on to check the spread of the disease (*Gazetta de Med. Zool.*).—*Veterinary Record*, October 26, 1901.

